# Post-exercise contractility, diastolic function, and pressure: Operator-independent sensor-based intelligent monitoring for heart failure telemedicine

**DOI:** 10.1186/1476-7120-7-21

**Published:** 2009-05-14

**Authors:** Tonino Bombardini, Vincenzo Gemignani, Elisabetta Bianchini, Emilio Pasanisi, Lorenza Pratali, Mascia Pianelli, Francesco Faita, Massimo Giannoni, Giorgio Arpesella, Rosa Sicari, Eugenio Picano

**Affiliations:** 1 Department of Echocardiography Lab, Institute of Clinical Physiology, National Council of Research, Pisa, Italy; 2 Digital Signal Processing Lab (DSPLAB), Institute of Clinical Physiology, National Council of Research, Pisa, Italy; 3 Department of Surgery and Transplants, University of Bologna, Italy

## Abstract

**Background:**

New sensors for intelligent remote monitoring of the heart should be developed. Recently, a cutaneous force-frequency relation recording system has been validated based on heart sound amplitude and timing variations at increasing heart rates.

**Aim:**

To assess sensor-based post-exercise contractility, diastolic function and pressure in normal and diseased hearts as a model of a wireless telemedicine system.

**Methods:**

We enrolled 150 patients and 22 controls referred for exercise-stress echocardiography, age 55 ± 18 years. The sensor was attached in the precordial region by an ECG electrode. Stress and recovery contractility were derived by first heart sound amplitude vibration changes; diastolic times were acquired continuously. Systemic pressure changes were quantitatively documented by second heart sound recording.

**Results:**

Interpretable sensor recordings were obtained in all patients (feasibility = 100%). Post-exercise contractility overshoot (defined as increase > 10% of recovery contractility vs exercise value) was more frequent in patients than controls (27% vs 8%, p < 0.05). At 100 bpm stress heart rate, systolic/diastolic time ratio (normal, < 1) was > 1 in 20 patients and in none of the controls (p < 0.01); at recovery systolic/diastolic ratio was > 1 in only 3 patients (p < 0.01 vs stress). Post-exercise reduced arterial pressure was sensed.

**Conclusion:**

Post-exercise contractility, diastolic time and pressure changes can be continuously measured by a cutaneous sensor. Heart disease affects not only exercise systolic performance, but also post-exercise recovery, diastolic time intervals and blood pressure changes – in our study, all of these were monitored by a non-invasive wearable sensor.

## Introduction

Telemonitoring heart failure patients is a very promising way of managing several complex and costly healthcare issues [1]. Recent European Society of Cardiology (ESC) guidelines on heart failure do recommend developing management programs for recently hospitalized patients with HF [2]. Screening left ventricular dysfunction would be very important, since heart failure is associated with high morbidity, mortality, and cost. Therefore, there is a great need for the design and implementation of an interactive real-time wireless telemedicine system [3]. Non-invasive new sensors for intelligent remote cardiac monitoring should be developed and integrated with other standard physiological sensor and biomarkers.

A new cutaneous force-frequency relation recording system has recently been validated in the stress echo lab, based on heart sound amplitude and timing variations at increasing heart rates. Expert monitoring of the heart – via a chest wall accelerometer – can reliably and non-invasively sense the contractile force and the filling function of the heart [4-6].

Information obtained from the ECG (QRS detector) and the heart sound vibration (HSV peak detector): heart rate, first heart sound and second heart sound, are analyzed by algorithms in order to derive the systolic force-frequency relation and the diastolic force-frequency relation. The physiological backgrounds are: 1) *The systolic force-frequency relation*. An increased heart rate progressively increases the contractile force of the heart [7]. In humans, an increase in heart rate from 60 to 170 bpm stimulates developed force. If chronic heart failure and/or myopathic, valvulopathic or ischemic cardiomyopathy is present, this intrinsic property of the myocardium is partially or totally depressed, because of which the contractile force decreases for cardiac frequencies of the order of 100 bpm or even lower [8]. 2) *The diastolic force-frequency relation*. If chronic heart failure is present, the decompensated myocardium undergoes a phenotypic change with activity alteration of the enzymes which regulate calcium homeostasis: diastolic uptake of calcium decreases and contractile performance improves only with bradycardia [7].

The daily life of both healthy subjects and patients involves periods of rest alternating with physical activity [9]; and activity consists of mild to strenuous exercise with obviously subsequent recovery periods. Testing this new sensor in the post-exercise period is mandatory before its application in telemedicine systems for close and continuous monitoring of heart failure in daily life.

The aims of this study were (1) to compare the sensor-based quantification with standard stress echo assessment in the post-exercise phase and (2) to exploit the sensor-based intelligent monitoring in a larger group of exercising subjects as a model of a wireless telemedicine system.

## Methods

### Patient selection

We enrolled 172 consecutive subjects referred for semi-supine exercise-stress echocardiography, mean age 55 ± 18 years. Subjects comprised 22 controls, 52 patients with CAD, 10 with dilated cardiomyopathy (DCM), 13 with hypertension, 18 with chronic obstructive pulmonary disease (COPD), 14 with valvular disease, 13 with corrected congenital heart disease and 30 with previous non-diagnostic exercise testing. Patient characteristics are summarized in Table 1. Coronary artery disease was defined by the presence of angiographically assessed coronary stenosis (with quantitatively assessed diameter reduction > 50% in at least one major coronary vessel) or previous myocardial infarction; patients with dilated cardiomyopathy had LV end-diastolic volume > 140 ml/m^2^, LV end-systolic volume > 70 ml/m^2^; 22 non-competitive athletes were the controls. The local Ethical Committee approved the study protocol. All patients gave their written informed consent before entering the study. All patients met the following inclusion criteria: 1) referred to stress echo for clinically-driven testing, 2) acoustic window of acceptable quality, and 3) willingness to enter the study, 3) recent (within 1 year) coronary angiography for all patients with stable chest pain syndrome or dilated cardiomyopathy. Exclusion criteria were: 1) unstable angina or recent myocardial infarction and 2) technically poor baseline echocardiographic examination. From the initial population of 178 patients, 6 were excluded due to a poor acoustic window (n = 4), or refusal to give written informed consent (n = 2).

**Table 1:** Patient characteristics

	Enrolled	Age	Gender (M/F)	LVEF %
Controls	22	40 ± 13	16/6	61 ± 8

CAD (no MI)	24	65 ± 8	17/7	62 ± 8

CHD (previous MI)	28	64 ± 9	23/5	50 ± 14

DCM	10	68 ± 9	8/2	39 ± 8

Hypertension	13	60 ± 7	8/5	61 ± 9

COPD	18	62 ± 12	15/3	61 ± 3

Valvular disease	14	59 ± 15	8/6	60 ± 11

Corrected congenital	13	28 ± 14	5/8	46 ± 32

Diagnostic	30	48 ± 16	18/12	58 ± 10

CAD = Coronary Artery Disease; MI = Myocardial Infarction; DCM = Dilated Cardiomyopathy; COPD = Chronic Pulmonary Obstructive Disease

### Semi-supine bicycle exercise

Graded semi-supine bicycle exercise echo was performed, starting at an initial workload of 25 watts lasting for 2 min; thereafter the workload was increased stepwise by 25 watts at 2-min intervals. A 12-lead electrocardiogram and blood pressure measurement were performed at baseline and every minute thereafter [10]. Two-dimensional echocardiographic monitoring was performed throughout and up to 5 min after the end of stress.

### Post-exercise

Standard echocardiographic data, echo-derived hemodynamic parameters and systemic pressure were measured at peak stress and at 1, 3 and 5 min post-exercise [11,12].

### Regional wall motion analysis

Regional wall motion analysis was evaluated at baseline and at peak stress with a semiquantitative assessment of a wall motion score index (WMSI), with the 17-segment model of the left ventricle, each segment ranging from 1 = normal/hyperkinetic to 4 = dyskinetic, according to the recommendations of the European and of the American Society of Echocardiography [13,14]. WMSI was derived by dividing the sum of individual segment scores by the number of interpretable segments [10,13]. Test positivity was defined as the occurrence of at least one of the following conditions: 1) new dyssynergy in a region with normal rest function (i.e. normokinesia becoming hypokinesia, akinesia or dyskinesia) in at least two adjacent segments [15,16]; 2) worsening of a resting dissynergy.

### Diagnostic end points and interruption criteria

The diagnostic end-points were the development of obvious echocardiography positivity. The test was also stopped, in the absence of diagnostic endpoints, for one of the following reasons for performing a submaximal, non-diagnostic test: intolerable symptoms, limiting asymptomatic side effects consisting of (a) hypertension (systolic blood pressure > 220 mmHg; diastolic blood pressure > 120 mmHg) (b) relative or absolute hypotension (> 30 mmHg fall in blood pressure), (c) supraventricular arrhythmias: supraventricular tachycardia or atrial fibrillation, (d) ventricular arrhythmias: ventricular tachycardia; frequent, polymorphous premature ventricular beats [10].

### Blood pressure analysis

One nurse recorded blood pressures of each patient at rest and during the study. The blood pressure recording was made using a sphygmomanometer and the diaphragm of a standard stethoscope [17]. Systolic and diastolic blood pressure was measured in the right arm. During exercise test and recovery, blood pressure was recorded with the patient lying in a left-rotated semi-supine position and gripping the left support with their left hand. Patients were told to let their right hand go limp when blood pressure was measured. Post-exercise hypotension was defined as at least 5 mmHg of diastolic blood pressure reduction vs the same exercise heart rate values [18].

### Echocardiographic hemodynamic assessment

By protocol, the first 52 consecutive enrolled subjects underwent both complete echocardiographic and sensor-based hemodynamic assessment at rest, peak exercise, and at the first, third, and fifth minute post-exercise (Table 2).

**Table 2:** Quantitative stress-echo assessment and calculated hemodynamic parameters

Measured values (rest, peak stress, recovery min. 1, 3, 5)	Method	Measure unit
Heart rate	ECG	*bpm*

LV ESV index	2D echo (Simpson rule)/BSA	*mL/m*^2^

LV EDV index	2D echo (Simpson rule)/BSA	*mL/m*^2^

SBP	Sphygmomanometer	*mmHg*

DBP	Sphygmomanometer	*mmHg*

**Calculated values** **(rest, peak stress, recovery min. 1, 3, 5)**		

Stroke volume index	EDV index – ESV index	*mL/m*^2^

Cardiac index	stroke volume index * heart rate	*L/min/m*^2^

Mean Arterial Pressure	(SBP-DBP)/3 + DBP	*mmHg*

LV elastance index	SP/ESV index	*mmHg/mL/m*^2^

Effective arterial elastance index (EaI)	(SBP*0.9)/Stroke volume index	*mmHg/mL/m*^2^

Ventricular-arterial coupling	LV elastance index/Eai	*ratio*

*SVR index*	80 * (MAP-5)/Cardiac index	dyne * sec * cm^5^

SBP = Systolic Blood Pressure; DBP = Diastolic Blood Pressure; SVR = Systemic Vascular Resistance, where 5 is an approximation of the right atrial pressure, and MAP is mean arterial pressure; Ventricular-arterial coupling is ventricular elastance/arterial elastance, which can be described as end-systolic pressure/end-systolic LV volume divided by end-systolic pressure/stroke volume. The pressure terms in the numerator and the denominator cancel out, and ventricular-arterial coupling equals stroke volume/end-systolic volume.

### Contractility, diastolic time, and arterial pressure measurements by precordial cutaneous sensor

**Figure 1:**
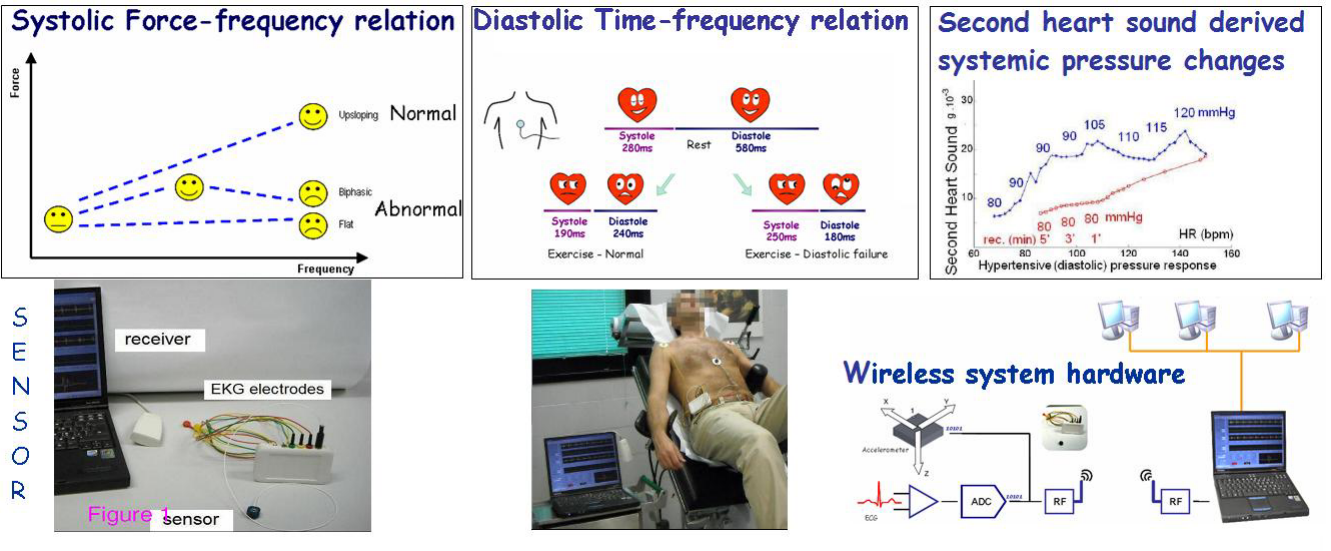
**Physiological backgrounds and the sensor**. *Upper panels, physiological backgrounds*. Left, the systolic force-frequency relation: an increased heart rate progressively increases the contractile force of the heart. In humans, an increase in heart rate from 60 to 170 bpm stimulates developed force. If chronic heart failure and/or myopathic, valvulopathic or ischemic cardiomyopathy are present, this intrinsic property of the myocardium is partially or totally depressed, due to which the contractile force decreases for cardiac frequencies of 100 bpm or even lower. Middle, the diastolic force-frequency relation: cardiac cycle abnormalities of patients with heart failure are characterized by a prolonged left ventricular systole and an abnormal shortening of left ventricular diastole. The systolic-diastolic mismatch is accentuated during exercise and may impair cardiac reserve in these patients by restricting ventricular filling and coronary perfusion. Right, the second heart sound (S2) amplitude recording simultaneously with diastolic blood pressure during stress: similar S2-frequency trend during stress (blue symbols) and recovery (red symbols) in a patient with exercise-induced diastolic hypertension and post-exercise hypotension. The S2 amplitude depends on the force with which the valves close, which in turn depends on the pressure gradient across the valve at the time of closure. *Lower panels, the sensor and the device*. A precordial non-invasive, operator-independent sensor and system for monitoring the systolic and diastolic force-frequency relation, and the pressure-frequency relation. Information obtained from the ECG (QRS detector) and the heart sound vibration (HSV peak detector) (heart rate, first heart sound and second heart sound) are analyzed by algorithms in order to derive the FFR. The data can be read remotely by a wireless sensor network (right).

**Figure 2:**
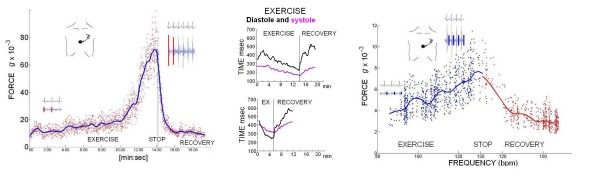
**Computing force variation as a function of heart rate**. Left panel, the systolic force-frequency relation. The amplitude of the vibration due to isovolumic myocardium contraction was obtained to record systolic force for each cardiac beat (red points). The curve of the systolic force variation as a function of heart rate was then computed; mobile mean (blue curve) was utilized to assess baseline, exercise, and recovery values. Middle, the curve of the systolic (pink line) and diastolic (black lines) time variation as a function of heart rate. Upper panel, a normal subject; lower panel, a patient with CHF shows prolonged systolic time with systolic/diastolic time reversal during exercise. At recovery, the systolic/diastolic time reversal is promptly normalized. Right panel, computing the second heart sound amplitude variation as a function of heart rate. All the parameters are acquired as instantaneous values during exercise (blue points) and recovery (red points); mobile mean (blue curve = exercise in progress; red curve = recovery) is recorded to assess arterial pressure changes.

**Figure 3:**
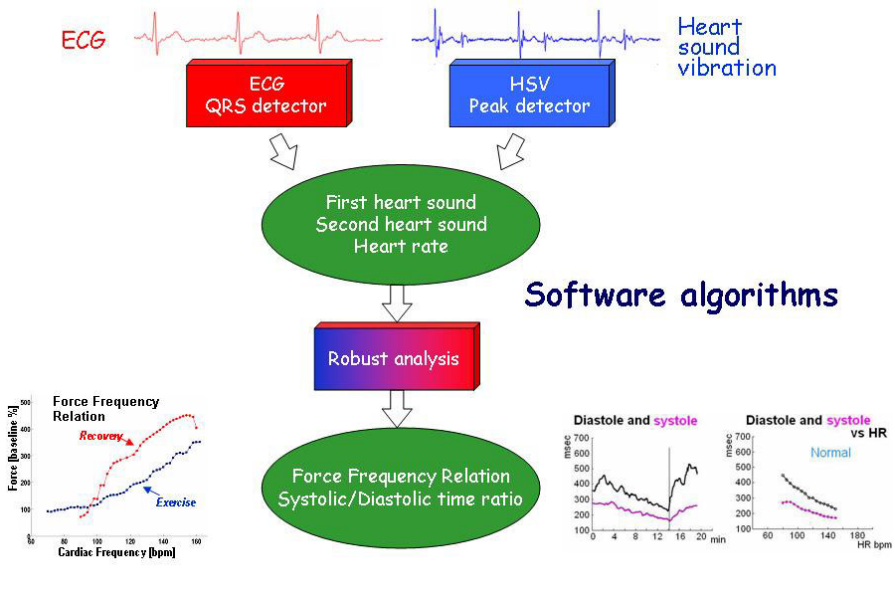
**Software algorithms**. The device provides a system comprising a microprocessor which receives informative signals from the heart regarding the patient's systolic and diastolic force-frequency relationship. The system records the points of the force-frequency relationship by successive beats, from which it then derives a diagram of the force-frequency relationship over predetermined time periods. If the point on the force-frequency relationship is within an abnormal region of the force-frequency relationship, specific provisions are adopted or an appropriate therapeutic regime is initiated. Monitoring the patient's condition can reveal an abnormal situation which although not critical in itself may predict a future worsening of chronic heart failure. In particular, it can help verify the effectiveness of therapies and their influence on the patient's condition in daily life. Positive or negative decompensation changes as a result of certain events can be determined, as well as variations in the force-frequency curve over 24 h. Thus a three-dimensional diagram is determined which for each heart rate not only indicates the instantaneous force value but also permits monitoring any variation of said value over time.

All the 172 enrolled subjects, scheduled for exercise stress, had standard ECG, pressure and WMSI evaluation and complete (both stress and recovery) sensor evaluation. The transcutaneous force sensor was based on a linear accelerometer of the LIS3 family (STMicroelectronics [Geneva, Switzerland]). The device includes in a single package a MEMS sensor that measures capacitance variation in response to movement or inclination and a factory trimmed interface chip that converts the capacitance variations into analog signals proportional to the motion. The device has a full scale of ± 2·*g *(*g *= 9.8 m/s^2^) with a resolution of 0.0005·*g*. We housed the device in a small case (Fig. 1) which was positioned in the mid-sternal precordial region and was fastened by a solid gel ECG electrode. The acceleration signal is acquired along with an ECG signal and transmitted to a laptop PC by wireless connection [19]. The data are analyzed using software developed in Matlab (The MathWorks, Inc Natick, Massachusetts, USA). A peak detector algorithm synchronized with the ECG scans the first 150 ms following the R-wave to record first heart sound force vibrations and the 100 ms following the T-wave to record second heart sound force vibrations (Fig. 2). These values are then filtered by a median filter, which averages the beat-to beat variations in the signal and removes outliers caused by movement artifacts. Contractility quantification through the first heart sound (FHS) amplitude recording and systemic arterial pressure changes through the second heart sound (S2) amplitude recording have been previously demonstrated [4,6]. Apart from the first and the second heart sound amplitude (related to the isovolumic contraction force and to the isovolumic relaxation force) this recording system was utilized to quantify both cardiological systole and diastole duration [5]. According to the physiological background, cardiological systole was demarcated by the interval between the first and the second heart sounds, lasting from the first heart sound to the closure of the aortic valve. The remainder of the cardiac cycle was automatically recorded as cardiological diastole [7] (Fig. 2). Information obtained from the ECG (QRS detector) and the heart sound vibration (HSV peak detector): heart rate, first heart sound and second heart sound are analyzed by algorithms in order to derive the systolic force-frequency relation, diastolic time-frequency relation and pressure-frequency relation [4-6]. The signals are displayed in real-time and processed by special-purpose software (Fig. 3). All the parameters were acquired at baseline, during stress and recovery; mobile mean was used to assess values. Values during exercise and recovery were computed as absolute value, and as percent changes vs baseline.

### Sensor-based intelligent monitoring as a model of a wireless telemedicine system

The systolic force-frequency relation, the diastolic time-frequency relation and the second heart sound derived systemic pressure were recorded at both stress and recovery in the 172 recruited subjects to exploit sensor-based dichotomy patterns in the leap to non-invasive intelligent remote heart monitoring.

### Statistical analysis

SPSS 11 for Windows was used for statistical analysis. The statistical analyses included descriptive statistics (frequency and percentage of categorical variables and mean and standard deviation of continuous variables). Since we observed large differences in baseline sensor-derived systolic force and S2 vibration amplitude, % changes were assessed during stress and recovery for interpatient and intergroup comparisons. Diastolic times were measured as absolute values simultaneously with systolic/diastolic time ratio. Chi-square test was used for comparisons between echo and sensor based contractility overshoot assessment (yes/no) and reduced pressure (yes/no) in the post- exercise phase. Changes in continuous variables during recovery were compared by analysis of variance for repeated measures. When this test was significant, individual comparisons of end-exercise value (recovery time = 0) and values during 1, 3 and 5 min of recovery were made by Duncan's multiple-range test. Since all the sensor-based parameters are physiologically heart-rate dependent, comparisons of recovery minutes 1, 3 and 5 values were made with sensor-recorded values at the same heart rates during exercise. A p-value < 0.05 was accepted as statistically significant.

## Results

### Stress echo results

Exercise time was 11 ± 5 min in the 22 controls and 9 ± 3 min in the 150 patients. WMSI was 1 at rest = peak stress in the 22 controls and 1.17 ± 0.35 at rest, 1.18 ± 0.37 peak stress in the 150 patients. Four patients had stress-induced ischemia (WMSI rest = 1.36 ± 0.35, peak = 1.77 ± 0.31).

### Comparison between sensor and echo assessment in 52 subjects

**Figure 4:**
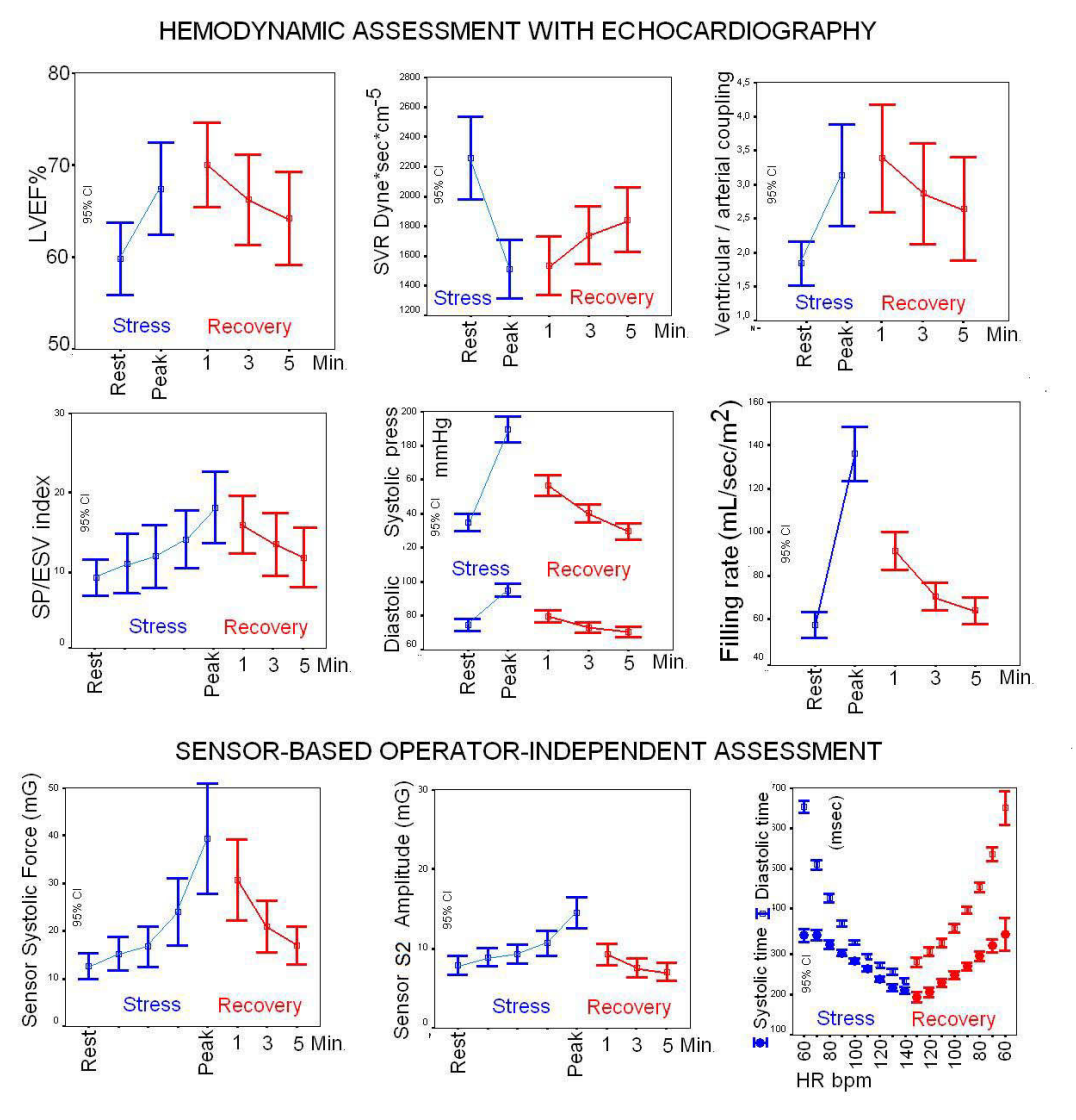
**Validation of sensor-based contractility, diastolic function and pressure assessment in the post-exercise period**. Upper panels, ecocardiographic quantitative hemodynamic changes during exercise in 52 subjects at rest (Watt 0), progressive graded bicycle exercise workload (blue symbols) and three stages of recovery (red symbols, R1, 3, 5 min). There was a significant increase (overshoot) in the ejection fraction and ventricular-arterial coupling during the first minute of recovery, compared with the end-exercise value. Minimal value of systemic vascular resistance is recognized at peak exercise, and during early recovery. Since contractility is physiologically heart rate-dependent (Bowditch Treppe or force-frequency relation), comparisons of recovery SP/ESV index values were made with exercise values recorded at the same heart rates: plasma catecholamine levels are still elevated during the early phase of recovery and a relatively slow decrease in contractility is observed. Systemic pressure measures were blunted in the post-exercise phase, due to nitric oxide spillover and adenosine accumulation. A transient, favourable mismatch between cardiac contractility and afterload reduction occurs at recovery in normal subjects, and to an even greater degree in diseased hearts. Lower panels, sensor-based data in the same 52 subjects. An effective, significant comparison with echocardiography is feasible for contractility (left, force-frequency relation) and blunted sensor-derived arterial pressure in the post-exercise phase (middle, S2 recording). Diastolic time during stress and diastolic time recovery overshoot monitoring is simple with the sensor, its difficulty comparable with echo measurement (operator-dependent and time-consuming). However, integration of sensor-times and echo-volume allows simple measurement of diastolic filling rate.

The first 52 consecutive enrolled subjects (7 controls) underwent both echocardiographic (Table 2) and sensor hemodynamic assessment (Fig. 4) at rest, peak exercise, and the first, third, and fifth minute of the post-exercise phase. At recovery minute 1 we observed a decrease in LV end-systolic volume, increased stroke volume index, and a decreased systemic diastolic and systolic pressure. Since contractility changes are measurable both with echo (SP/ESV index) and with the sensor (FHS vibrations amplitude), comparisons were made. Heart rate dropped rapidly at recovery, but at each recovery step contractility was higher than exercise values in 42 subjects with echo and in 40 with sensor (Chi-square p < 0.01). Since systemic pressure changes are measured by protocol and derivable by the sensor (S2 vibrations amplitude), comparisons were made (Fig. 4, lower panels); 41 (80%) of the subjects had post-exercise hypotension, and 36 (69%) second heart sound undershoot (Chi square p < 0.01). Diastolic time length was easily measured only by the sensor: at each recovery heart rate diastole was longer than during exercise (Fig. 4).

### Sensor-based force-frequency relation, diastolic time-frequency relation and derived systemic pressure in the post-exercise phase in the 172 enrolled subjects

**Figure 5:**
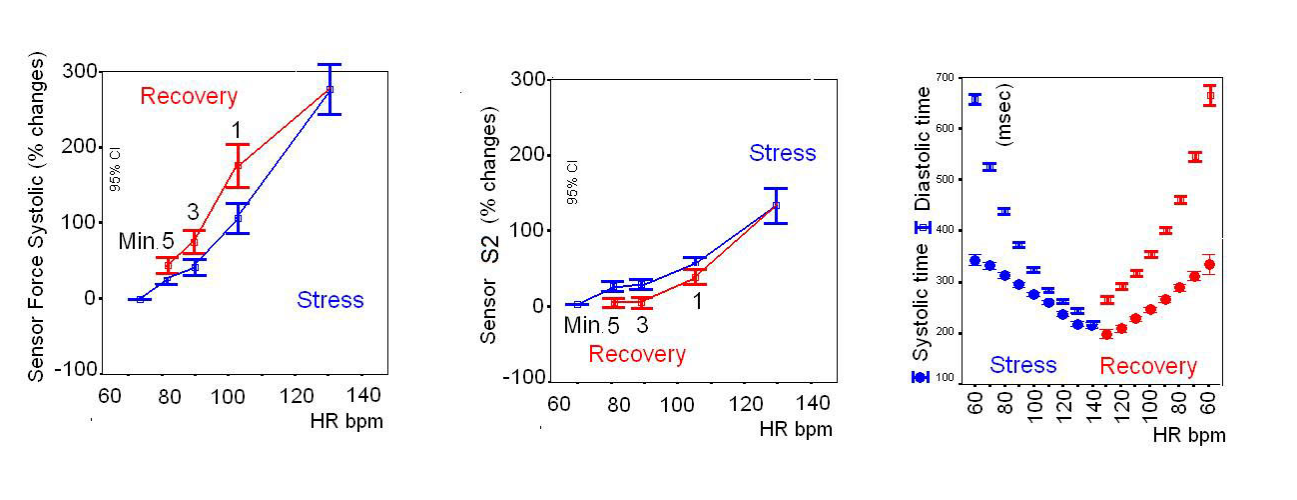
**Cumulative sensor data in the 172 enrolled subjects**. Progressive graded exercise workload (blue symbols) and three stages of recovery (red symbols, R1, 3, 5 min). Left panel, contractility at different heart rates (force-frequency relation): higher mean force data are observed at each recovery step with respect to exercise values. Middle panel, sensor-derived systemic pressure changes: lower mean pressure values are observed at each recovery step in comparison with exercise values. Right panel, diastolic (empty symbols) and systolic (full symbols) times at increasing heart rates during exercise (blue) and at decreasing heart rates during recovery (red): diastolic times overshoot is observed at each recovery heart rate, with improved ventricular filling and coronary perfusion time.

**Figure 6:**
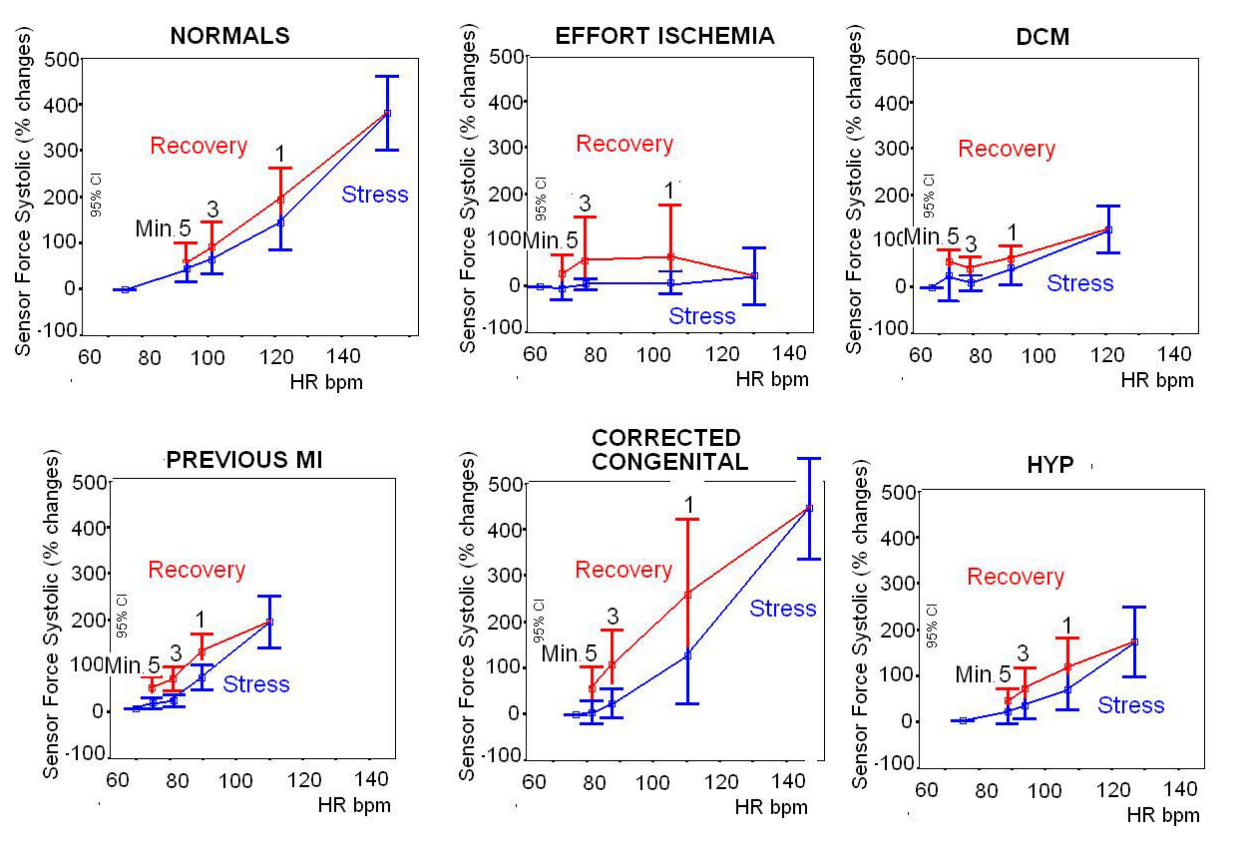
**Cumulative sensor data in the controls and different patient groups**. Progressive graded exercise workload (blue symbols) and three stages of recovery (red symbols, R1, 3, 5 min). According to physiological background, FFR is steeper in controls vs DCM, previous MI, and hypertensive patients. A recovery contractile overshoot (with a relative increase of recovery contractility of more than 10% with respect to the exercise value) was more frequent in patients vs controls. Patients with stress-induced ischemia (upper middle panel) showed a flat contractile reserve at ischemia and a clear recovery contractile overshoot.

Sensor data for contractility, diastolic time-frequency relation, and second heart sound were obtained in all the 172 enrolled subjects (feasibility = 100%) and are displayed in Fig. 5. Contractility comparisons between different groups of patients based on the incoming disease are reported in Fig. 6.

### The post-exercise force frequency relation and the contractile overshoot

**Figure 7:**
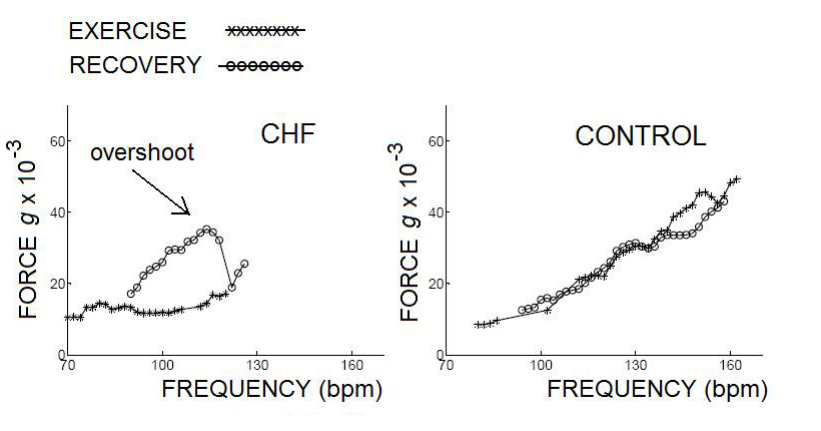
**Post-exercise contractility overshoot**. Left, the exercise force-frequency relation is blunted in a CHF patient. Post-exercise contractility overshoot (defined as a relative increase in recovery contractility of more than 10% with respect to the exercise value) was recorded with the cutaneous wireless sensor. Right, the exercise force-frequency relation is upsloping in a control subject. Post-exercise contractility curve mirrored the stress values. Cross symbols = exercise; empty symbols = recovery.

During exercise the force frequency relation was upsloping in controls and blunted in patients. Post-exercise contractility overshoot (defined as a relative increase in recovery contractility of more than 10% with respect to the exercise value) was more frequent in patients than controls (27% vs 8%, p < 0.05) (Fig. 7). The overshoot phenomenon was found in 2 controls and 45 patients (p < 0.05), and more frequently in DCM (50%), congenital (69%), hypertensive (39%), previous MI (36%) vs CHD (21%) or COPD (22%) or diagnostic (17%) patients, chi-square p = 0.003.

### Diastolic time-frequency relation and the post-exercise diastolic time overshoot

**Figure 8:**
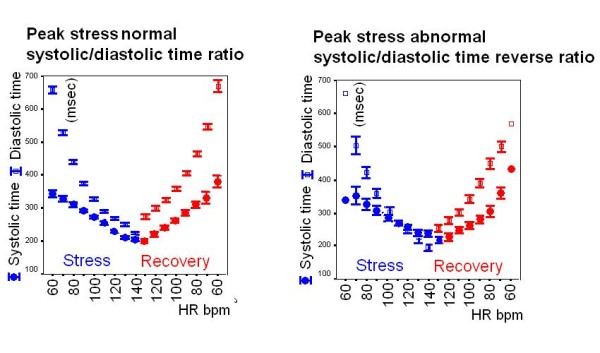
**Sensor-based diastolic time-frequency relation in the post-exercise phase**. Diastolic (empty symbols) and systolic (full symbols) times at increasing heart rates during exercise (blue) and at decreasing heart rates during recovery (red). At peak stress 109 subjects showed a normal diastolic time still longer than systole (left panel). At peak stress (right panel), 63 subjects showed a reversal of the systolic/diastolic ratio, with the duration of systole longer than diastole. The systolic-diastolic mismatch, with relative systolic dominance, was promptly resolved during recovery. At each recovery heart rate the diastolic time increased with respect to the exercise period in both groups, with a recovery diastolic time overshoot. Diastolic time overshoot is observed at each recovery heart rate, with improved ventricular filling and coronary perfusion time.

From rest to peak exercise, the mean systolic time was shortened by 25 ± 11%. The diastolic time decreased more markedly during exercise (by 53 ± 13% at peak stress) [5]. At 100 bpm heart rate during exercise, 20 patients (and at peak stress, 63 subjects) showed a reversal of the systolic/diastolic ratio, with the duration of systole longer than that of diastole (Fig. 8). The diastolic time increased abruptly during the first minute of recovery, with an overshoot phenomenon. At each recovery heart beat frequency, the diastolic time was higher than the diastolic time recorded during exercise, all p < 0.05 vs exercise. At 100 bpm heart rate during recovery, only three patients still showed reversal of the systolic/diastolic ratio, with the duration of systole longer than diastole (p < 0.05 vs exercise). In the post-exercise period, the sensor-based quantification of the diastolic time-frequency relation (diastolic time at each value of decreasing heart rate) showed that diastole lengthened in the post-exercise phase in both controls and patients: at recovery 100 bpm heart rate, + 39 ± 22 msec lengthening in controls vs + 33 ± 21 msec lengthening in patients, p = ns. However, at the individual level a broad spectrum was found in patients with a -30 msec to + 81 msec range.

### Sensor-derived systemic pressure and post-exercise hypotension

**Figure 9:**
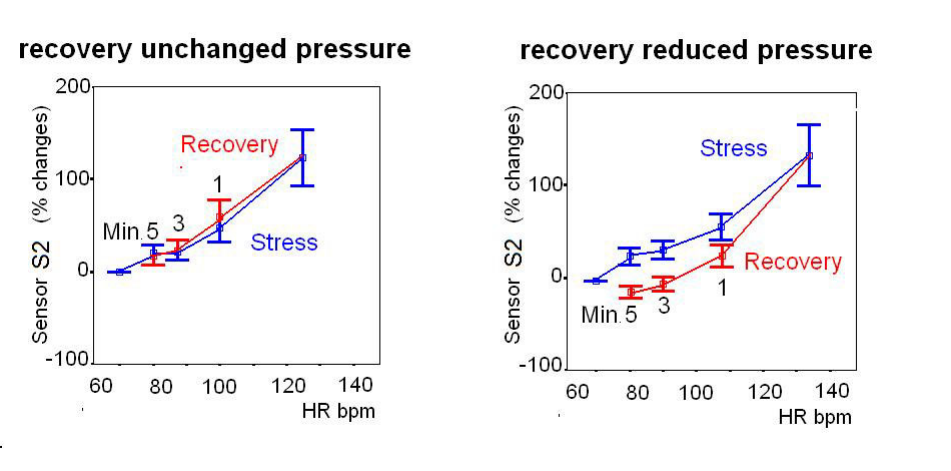
**Sensor-derived systemic pressure and post-exercise hypotension**. Cumulative sensor data in subjects without (left panel) and with (right panel) post-exercise hypotension. Progressive graded exercise workload, blue symbols; three stages of recovery, red symbols, R1, 3, 5 min. A significant correlation was found between post-exercise hypotension and recovery S2 undershoot.

A significant correlation was found between post-exercise hypotension and recovery second heart sound amplitude (S2) undershoot: 138 subjects had normal post-exercise hypotension; 83% of the subjects with post-exercise hypotension had S2 undershoot in the recovery, while 84% of the 34 subjects without post-exercise hypotension had stable rate-S2 curve at recovery (Fig. 9).

## Discussion

### Comparisons between sensor- and echo-derived information on function during recovery

At force-frequency analysis, with both computed elastance (SP/ESV index) and operator-independent sensor (FHS vibrations amplitude) most patients showed a contractility increase at recovery. Standard systemic pressure measures (obtained by sphygmomanometer) were blunted in the post-exercise phase, and a general decrease in the sensor-based derived systemic pressure were found [6]. Furthermore, during recovery as during stress, the cutaneous operator-independent force sensor described systolic and diastolic duration in real time. Simultaneous calculation of stroke volume with echo and diastolic time with force sensor allowed us to monitor the diastolic filling rate [5].

### Sensor-based post-exercise force-frequency relation in normal and diseased hearts

During exercise the normal force-frequency relation is upsloping, while a flat-biphasic force-frequency relation is abnormal [20-24]. A post-exercise sensor-based contractility overshoot was more frequent in patients vs controls, and frequently associated with an abnormal blunted force-frequency relation during exercise. Several investigators using different methods [12,25-27] have reported an overshoot of cardiac function during recovery from maximal exercise in patients with cardiac disease. Koike et al. [26] have shown a marked rebound of stroke volume immediately after cessation of upright exercise in patients with previous myocardial infarction. Tanabe at al. [27] found an overshoot of cardiac output at 1 min of recovery in patients with severe CHF, along with poor cardiac output response to exercise. They found that not only O_2 _uptake but also cardiac output fell much more slowly after maximal exercise as CHF worsened. Although insufficient afterload reduction during exercise in CHF contributes to the impaired stroke volume response to exercise, systemic vascular resistance at 1 min of recovery significantly decreased from that at peak exercise in patients with CHF who showed overshoot of cardiac output during recovery [28-31]. The marked increase in stroke volume during early recovery in patients with overshoot appears to result from both an immediate afterload reduction and a relatively slow decrease in cardiac sympathetic stimulation during recovery [32,33]. Knowledge of the post-exercise overshoot also had prognostic value since patients with a moderate exercise intolerance and a normal recovery period had a better prognosis than a patient with a post-exercise overshoot [34].

### Sensor-based diastolic time-frequency relation in the post-exercise

We found that at each recovery heart beat frequency, the diastolic time was higher than the diastolic time recorded during exercise, in both controls and patients. Why does diastolic time increase in the post- exercise phase? The total cardiac cycle duration is algebraically dependent on the heart rate [= 60,000 msec/heart rate] [35]. However at each heartbeat frequency the fixed total cardiac cycle time can be differently divided between systole and diastole [5]. The diastolic time fraction is determined by factors that modulate systolic duration through modulation of myocyte contraction [36-40]. At recovery, improved myocardial contractility and reduction in systemic vascular resistance significantly shortened left ventricular ejection time, with a proportionate increase in diastolic time fraction. Stress-induced "systolic-diastolic mismatch" can be easily quantified by a disproportionate decrease in diastolic time fraction, and is associated with several cardiac diseases, possibly expanding the spectrum of information obtainable during stress [5]. The post-exercise diastolic time fraction indicates the duration of absence of compression of intramural vessels during a heartbeat and has a dominant role in the subendocardial layer – whose perfusion is mainly diastolic, whereas the perfusion in the subepicardial layer is also systolic [36,38]. The lengthening of cardiological diastole is much more pronounced than lengthening of cardiological systole, and the former is much more effective for subendocardial perfusion, even in the absence of coronary artery disease. This indicates the relevance of monitoring both exercise and recovery diastolic time in the critically diseased heart [37,39].

### Systemic pressure in the post-exercise phase and sensor-monitored pressure changes

A significant correlation was found between post-exercise hypotension and recovery S2 undershoot (Fig. 9). In this investigation, blood pressure (systolic, diastolic and mean) correlated closely with S2 amplitude during both stress and recovery [6]. This could be explained by the fact that amplitude is primarily determined by one factor, the force of valve closure [41,42]. In the selected patients of our study, a significant correlation was found between post-exercise hypotension and recovery second heart sound lower amplitude, to confirm the sensor's ability to mirror the diastolic pressure trend. Post-exercise hypotension has been demonstrated in both hypertensive and healthy subjects [43], and it has been attributed to a decrease in cardiac output and/or systemic vascular resistance [44-47]. Acute exercise may serve as a non-pharmacological aid in the treatment of hypertension. S2 amplitude monitoring could be a method for assessing the efficacy of acute post-exercise blood pressure reduction.

### Clinical implications, implantable vs. wearable sensors and chronic heart failure

Congestive heart failure (HF) is a serious public health problem due to its prevalence, high mortality, high morbidity, and the expense of ongoing therapy [2].

Several strategies to control fluid volume status are used in the practice. Clinic visits for assessment of filling pressure by physical examination, multiple types of non-invasive measurements, and repeated cardiac catheterization may be employed. There is considerable cost and inconvenience for the patient associated with these strategies and, more importantly, these methods represent pressure and volume status only as one discrete point in time without the perturbance of daily activities or stress. A system of frequent monitoring could alert clinicians to early signs and symptoms of decompensation, providing the opportunity for intervention before patients become severely ill and require hospitalization [1].

#### Implantable hemodynamic monitors (IHMO)

Implantable hemodynamic monitors that are capable of measuring chronic right ventricular oxygen saturation and pulmonary artery pressure are currently being developed (Chronicle, Medtronic Inc. Minneapolis, Minnesota, USA) [48].

Cardiac resynchronization therapy/defibrillators and implantable cardioverter defibrillators with continuous intrathoracic impedance monitoring capabilities (OptiVol fluid status monitoring; Medtronic Inc. Minneapolis, Minnesota, USA) have recently been introduced and may provide an early warning of thoracic fluid retention [49]. However the predictive values of these implantable devices is still unknown [48,49]. Furthermore, such strategies will have to be evaluated for cost effectiveness, scalability, safety, and acceptability to patients.

#### Wearable sensors

As technologies such as micro-technologies, telecommunication, low-power design, new textiles, and flexible sensors become available, new user-friendly devices can be developed to enhance the comfort and security of the patient. Since clothes and textiles are in direct contact with about 90% of the skin surface, smart sensors and smart clothes with non-invasive sensors are an attractive solution for home-based and ambulatory health monitoring [50]. All these systems can provide a safe and comfortable environment for home healthcare, preventive medicine, and public health.

#### The systolic and diastolic FFR sensor

Expert monitoring of the heart – via a chest wall sensor – can reliably and non-invasively sense the contractile force and the diastolic function of the heart. Dichotomy patterns: upsloping vs flat FFR, stress-induced "systolic-diastolic mismatch (yes/no), post-exercise contractility overshoot (yes/no), post-exercise diastolic time overshoot (yes/no), post-exercise normal hypotension (yes/no) are easily recognized by sensor-based intelligent monitoring (Table 3). Monitoring the patient's condition can reveal an abnormal situation which although not critical in itself may predict a future worsening of chronic heart failure. In particular, it can help verify the effectiveness of therapies and their influence on the patient's condition in daily life. Positive or negative decompensation changes as a result of certain events can be determined, as well as variations in the force-frequency curve over a 24-h period. When abnormal patterns are recognized, specific provisions are adopted or an appropriate therapeutic regime is initiated [51]. This novel method and device for the diagnosis and therapy of chronic heart failure can be integrated with other standard physiological sensors and biomarkers.

**Table 3:** Sensor-monitored force-frequency relation in normal and diseased hearts

Clinical status	Force-Frequency Relation (FFR)	Diastolic time-frequency relation	S2-frequency relation
Normal	Upsloping FFR	systolic/diastolic time ratio < 1	Normal upsloping
	Normal recovery	Normal recovery	Recovery undershoot
Acute ischemia	Acute biphasic FFR	Acute systolic/diastolic time ratio > 1	Acute S2 blunting
	Recovery overshoot	Recovery overshoot	Recovery overshoot
CHF worsening ↓	1- Blunted FFR slope		
↓↓	2- From upsloping to biphasic FFR	Systolic/diastolic time ratio > 1 at lower HR	S2 blunting
↓↓↓	3- Lower critical HR in biphasic FFR		
	- Recovery overshoot	Recovery overshoot	
CHF improving ↑↑↑	3- Upsloping FFR		
↑↑	2- From biphasic to upsloping FFR	Systolic/diastolic time ratio > 1 at higher HR	Upsloping S2
↑	1- Higher critical HR in biphasic FFR		
	- Normal recovery		Upsloping S2
Hypertension/diastolic failure	Blunted FFR	Systolic/diastolic time ratio > 1 at lower HR	Steeper S2 curve
	Recovery overshoot	Recovery overshoot	Recovery overshoot

Atrial fibrillation	Preceding and pre-preceding interval FFR dependence	Systolic/diastolic time ratio scattering	S2 scattering

S2 = Second heart sound peak amplitude vibrations; HR = Heart Rate; FFR recovery overshoot = a relative increase in recovery FFR of more than 10% with respect to the exercise value

Our research will continue to optimize features of both the sensor and algorithm, and will develop an engineering model for industrialization, aiming at the device's eventual use in long-term home monitoring for tailoring drug treatment and preventing re-hospitalization.

## Conclusion

Contractility can be continuously measured by a cutaneous accelerometer during the post-exercise phase. Knowledge of the recovery overshoot phenomenon could be helpful for recognizing advanced failing patients in home monitoring systems. Diastolic duration time can be monitored during the post-exercise phase and a recovery diastolic time overshoot phenomenon can be easily assessed by the sensor. S2 amplitude monitoring is a method for assessing acute post-exercise blood pressure reduction. The daily life of both healthy subjects and patients involves periods of rest alternating with physical activity; and activity consists of mild to severe exercise with obviously subsequent recovery periods. Heart disease affects not only peak exercise systolic performance, but also post-exercise recovery, diastolic time intervals and blood pressure changes – all of which can be monitored by a non-invasive wearable sensor.

## Abbreviations

BSA: body surface area; DBP: diastolic blood pressure; CAD: coronary artery disease; CHF: congestive heart failure; CO: cardiac output; DCM: idiopathic dilated cardiomyopathy; EaI: effective arterial elastance index; EDV: end-diastolic volume; EF: ejection fraction; ESV: end-systolic volume; FFR: force-frequency relation; FHS: first heart sound; *g*: acceleration unit (9.8 m/sec2); HF: heart failure; HIMO: implantable hemodynamic monitor; HR: heart rate; S2: second heart sound; SBP: systolic blood pressure; SVR: systemic vascular resistance; WMSI: wall motion score index

## Competing interests

The authors declare that they have no competing interests.

## Authors' contributions

TB designed this study, performed the data analysis, and drafted the manuscript; EPa, LP and MP were responsible for data collection and revised the manuscript; VG, EB, FF and MG were responsible for technology development and digital signal processing; GA and RS contributed to data discussion; EPi contributed to preparation of study design, data discussion, and critical revision of the manuscript.
